# Huntington’s Disease: Calcium Dyshomeostasis and Pathology Models

**Published:** 2017

**Authors:** Y.A. Kolobkova, V.A. Vigont, A.V. Shalygin, E.V. Kaznacheyeva

**Affiliations:** Institute of cytology of the Russian Academy of Sciences, Tikhoretsky ave. 4.,Saint-Petersburg, 194064 , Russia

**Keywords:** calcium, induced pluripotent stem cells (HD-iPSCs), huntington’s disease, neurodegeneration, huntingtin, SOC

## Abstract

Huntington’s disease (HD) is a severe inherited neurodegenerative disorder characterized by motor dysfunction, cognitive decline, and mental impairment. At the molecular level, HD is caused by a mutation in the first exon of the gene encoding the huntingtin protein. The mutation results in an expanded polyglutamine tract at the N-terminus of the huntingtin protein, causing the neurodegenerative pathology. Calcium dyshomeostasis is believed to be one of the main causes of the disease, which underlies the great interest in the problem among experts in molecular physiology. Recent studies have focused on the development of animal and insect HD models, as well as patient-specific induced pluripotent stem cells (HD-iPSCs), to simulate the disease’s progression. Despite a sesquicentennial history of HD studies, the issues of diagnosis and manifestation of the disease have remained topical. The present review addresses these issues.

## INTRODUCTION

The inherited nature of Huntington’s disease (HD) was discovered and described by George Huntington in his original paper almost a century and a half ago [1]. HD has an autosomal dominant type of inheritance and is caused by a mutation that leads to an increased number of CAG-repeats in the huntingtin (Htt) protein gene localized on chromosome 4p16.3. This mutation increases the number of glutamine (Q) residues in the N-terminal region of Htt, which, in different ways, leads to the observed pathologies [2]. Normally, the polyglutamine tract contains no more than 35 glutamines [3]. Huntington’s disease is characterized by selective death of GABAergic striatal neurons [3], while dopaminergic neurons of the substance nigra are what are mainly affected in Parkinson’s disease [4], and preferential loss of hippocampal neurons occurs in Alzheimer’s disease [5]. To date, several mechanisms are believed to contribute to the pathogenesis of HD, including the new toxic properties of mutant Htt (mHtt), concomitantly with the dysfunction of normal Htt [6]. These changes lead to a dysregulation of the transcription of the gene encoding Htt [7], synaptic dysfunction and excitotoxicity [8, 9], mHtt dyshomeostasis [10], intracellular transport defects [11], mitochondrial dysfunction [12-14], and calcium signaling disturbances [15-17].

## MANIFESTATION AND DIAGNOSIS OF HD

The prevalence of HD is quite high: the disease incidence rate is approximately 1 per 1,000,000 people of Asian and African descent and 5–10 per 100,000 Caucasians, besides the many people who are at risk. HD is more common in males than in females, manifests itself primarily at age older than 30 years, and usually leads to death 15–20 years after the onset of the first symptoms. At the same time, long polyglutamine tracts may be the cause of juvenile or even infantile HD. Mutations increasing the length of glutamine repeats up to 36–40Q are associated with incomplete penetrance; if repeats are longer than 41Q, the disease is fully penetrant [18].

The polyglutamine tract length of mHtt directly correlates with the disease’s severity and in most cases inversely correlates with the age of onset of the first symptoms [19]. However, there is a significant variability between the expected and actual age of manifestations [20]. For example, for the same length of polyglutamine tract, especially in the range of 40–44Q, the age of manifestations may differ by 20 years [21]. This difference may be explained by the presence of some genetic modifiers that regulate the expression of both Htt and other proteins and, thereby, mediate increased sensitivity or resistance to the disease. For example, the S18Y polymorphism in the gene encoding ubiquitin C-terminal hydrolase L1 is associated with late manifestations of HD [22]. In patients with the M441T mutation in the gene encoding the Htt-associated protein (Hap1), HD manifested itself at an earlier age due to a weakened interaction between Hap1 and mHtt and, thereby, increased Htt-mediated toxicity [23]. Recently, a single nucleotide polymorphism in the NF-κB binding site located in the *Htt *gene promoter was shown to reduce the promoter activity and, as a consequence, Htt expression, which led to late manifestations of HD [24].

However, a genetic mutation is not sufficient for both predicting the individual risk to a disease and assessing the current physiological processes in a body. Therefore, identification of biomarkers of HD progression, which may indicate pathological processes before the manifestation of clinical symptoms, is important for the development of new drugs and evaluation of treatment efficacy and the effect of environmental factors. Recent advances in the diagnosis include quantification of the mHtt level by a hypersensitive immunological analysis of single molecules in the cerebrospinal fluid samples of subjects with a mutation in the gene [25].

Because Htt is expressed in almost all body tissues, mHtt-induced changes can be detected even in the blood. Involvement of leukocytes in the immune response makes a blood test an ideal method to identify pathological processes, such as peripheral inflammation, in HD. In HD, expression of the *H2A histone family, member Y *gene is increased in the blood [26]. Clinical trials demonstrated that the expression of this gene both in the blood samples and brain tissues of HD patients was 1.6-fold higher than that in controls. Next-generation sequencing and Fluidigm technologies were used to identify five genes that encode the potential HD biomarkers detected in the blood of patients [27]. A correlation between cognitive impairment in HD and the levels of the peptide hormone prokineticin 2 (PROK2) involved in the regulation of circadian rhythms was revealed [28]. Therefore, PROK2 is considered as one of the promising markers of HD progression. Also, an elevated level of aquaporin 9 mRNA was detected in the blood of HD patients [29].

The variability of the clinical HD phenotype and the potential effect of some environmental and pharmacological factors lead to the need to combine different markers of HD progression. A decreased level of N-acetylaspartate (NAA) in brain tissues is considered a reliable indicator of neuronal dysfunction and death and can be measured noninvasively by MRI, which is important for a clinical diagnosis [30]. The NAA level in patients with early HD manifestations is lower than that in a control group. At the same time, the level of a gliosis marker, myo-inositol, is significantly increased in these patients [31]. A relationship between the NAA level and the disease severity opens the opportunity to use this metabolite as an identifier of neurochemical reactions in evaluating the effectiveness of potential therapeutic agents.

In HD, there is an increase in the serum concentrations of vasopressin that play an important role in the homeostasis of body fluids [32], 8-hydroxy-2-deoxyguanosine (an indicator of oxidative DNA damage), and lipid peroxidation products (lactic acid, 4-hydroxynonenal, and malondialdehyde), which makes these compounds potential biomarkers [33]. Reduced levels of glutathione peroxidase and Cu,Zn-superoxide dismutase were detected in the erythrocytes of HD patients [34], and elevated levels of cytokines, including interleukins 4, 6, 8, 10, and 23, TNF-Α, as well as clusterin, were found in postmortem brain sections and plasma samples [35].

The use of all these biomarkers will provide an accurate assessment of the efficacy of new treatments and increase the safety and efficacy of preclinical and clinical trials.

## HUNTINGTIN PROTEIN

**Fig. 1:**
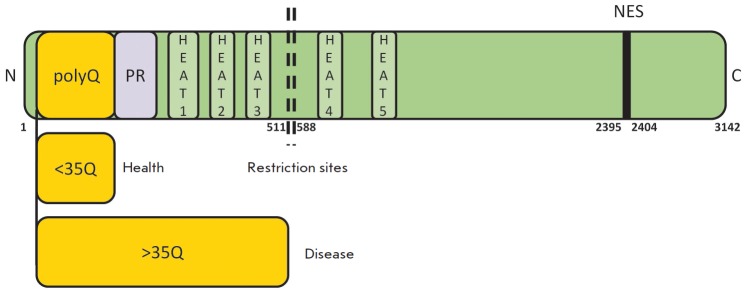
The domain structure of human huntingtin: PolyQ – polyglutamine tract; PR – proline-rich domain responsible for protein-protein interactions; HEAT repeats; protease cleavage region; NES – nuclear export signal.

The development of HD is associated with a mutation in the huntingtin protein gene. Huntingtin is a protein with a molecular weight of about 350 kDa and a polyglutamine tract at the N-terminus. In the same region, there is a proline-rich domain involved in protein-protein interactions and protecting huntingtin from aggregation [36]. *Fig. 1* presents the domain structure of human huntingtin.

In the cell, huntingtin functions as a scaffold protein; i.e., it provides colocalization of the proteins interacting with it, helping them to perform their functions. Huntingtin (especially its N-terminal region) interacts with numerous proteins, performing a wide variety of functions ranging from vesicular transport and endocytosis to the regulation of transcription and apoptosis [37].

The huntingtin molecule looks like a solenoid with a hydrophobic core composed of docked HEAT repeats. These repeats, together with the proline-rich region, participate in protein-protein interactions. The name HEAT is an acronym for four proteins in which the repeat structure was first identified (huntingtin, elongation factor 3, PR65/A (a phosphatase 2A subunit), and lipid kinase TOR) [38]. The structure of the short N-terminal fragments of huntingtin was studied by X-ray diffraction [39] and nuclear magnetic resonance [40]. Recently, the secondary structure of huntingtin was shown to correlate with the length of the polyglutamine tract [41]. Images of normal and mutant huntingtins having a spherical structure with a cavity were obtained by electron microscopy [41]. Images of Htt23Q and Htt78Q are very similar, but the impact of the polyglutamine tract on the huntingtin structure suggests that huntingtin undergoes dramatic conformational changes upon interaction with its binding partners [41]. Despite these facts, it is not yet fully understood how the structure of huntingtin is related to its functions, and how mutation-induced changes in its structure lead to the observed pathologies.

HD is believed to be associated with cleavage of the N-terminal fragment from mutant huntingtin, which is encoded by the first exon and contains the polyQ tract. The cleaved fragment accumulates in the nucleus, while wild-type huntingtin is localized mainly in the cytosol [42, 43]. Posttranslational modifications of huntingtin control its localization [44]. Accumulation of aggregated N-terminal mHtt fragments and associated proteins, such as various transcription factors, heat shock proteins, and proteasome components, in the nucleus complicates their functioning and, as a consequence, leads to various cell pathologies [45].

Neuropathological markers of HD include intracellular inclusions formed by N-terminal mHtt fragments, which were found in a postmortem study of the brains of HD patients, as well as in animal and cell models of HD [42, 46, 47]. The formation of insoluble aggregates in HD leaves no doubt, but many studies have demonstrated that this process is not directly associated with neuronal degeneration. For example, expression of mHtt in a striatal neuron culture demonstrated an accumulation of insoluble protein aggregates, which did not correlate with neuronal death. Furthermore, a decrease in intranuclear inclusions of mHtt coincided with an aggravation of neurodegenerative processes [48]. A study of neurons expressing the first exon of *mHtt *also showed that neuronal death correlates with an increase in the polyglutamine tract length and with the amount of diffuse mHtt in the cell, while accumulation of aggregates just reduces the level of dissolved mHtt, thereby increasing the survival of neurons [49]. It is believed that unstable heterogeneous prefibrillar aggregates are responsible for amyloid toxicity, whereas mature fibrils are stable and harmless reservoirs of toxic species [50].

These facts suggest that the formation of aggregates in HD cannot be the sole cause of pathology development, and elucidating the molecular basis of HD remains a topical issue.

## HD MODELING

**Fig. 2:**
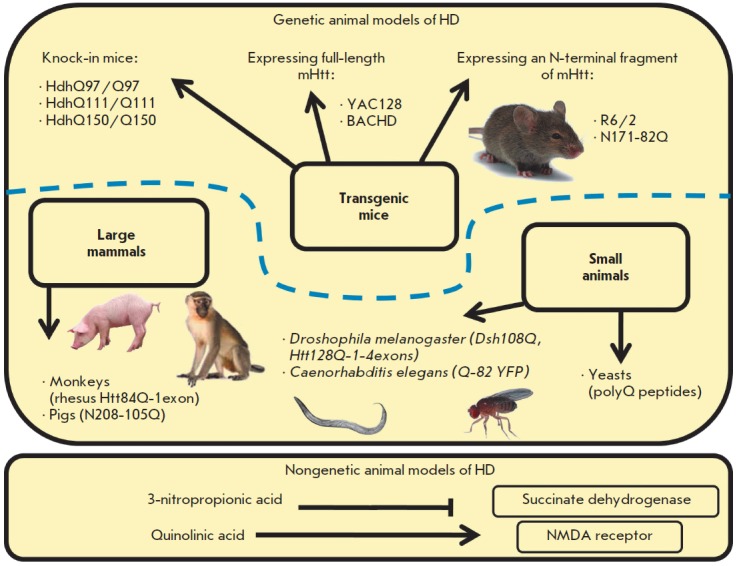
Animal models of Huntington’s disease

Generation of adequate disease models is very important for studying the molecular mechanisms of neuro degeneration and searching for new drugs. Since HD is a hereditary disease caused by a mutation in a single gene, genetic manipulations can be used to create various models that accurately simulate the disease (*Fig. 2*).

R6/2 mice have a stable phenotype that includes impaired coordination and gait, hypoactivity, and cognitive dysfunction. The disease manifestation age in this model is about 4 weeks [51]. R6/2 mice were detected with aggregates containing intracellular inclusions similar to those found in the biopsy specimens of the brain tissues of HD patients [52]. However, despite the stability of the phenotype, R6/2 mice cannot be an accurate HD model, because they express only the N-terminal fragment of a mutant protein. Nevertheless, R6/2 mice are widely used to simulate the common features of polyglutamine diseases, including the abnormal protein conformation due to an expanded polyQ tract.

YAC128 and BACHD transgenic mice containing 128Q and 97Q in a full-length mutant protein, respectively, have a milder HD phenotype compared to that of the R6/2 model [53].

Mouse knock-in models have the weakest HD phenotype. Even upon 150Q expression, HdhQ150/Q150 mice had fewer abnormalities than R6/2 mice. In HdhQ150/Q150 mice, the first disease symptoms, including motor dysfunction and gait disturbances, developed at a later age [54].

Despite the fact that mouse models are based on a disease-inducing mutation, most of them lack the stable neuronal loss that occurs in patients. To overcome this problem, other model organisms are required. Toxicity of the N-terminal mHtt fragment is more pronounced in large mammals, such as pigs and monkeys, while sheep expressing full-length mHtt lack marked phenotypic signs of the disease [55]. However, despite a number of advantages, these models have serious drawbacks, such as high cost and the need for specialized laboratory animal care equipment.

*Drosophila melanogaster *and *Caenorhabditis elegans *are also used to model HD. The advantage of these organisms is a short lifespan and rapid reproduction. Identification of a human Htt ortholog in *Drosophila *suggests that these insects have the pathways necessary for the normal functioning of Htt, which makes *Drosophila *a good model for studying HD [56]. Another interesting feature of *Drosophila *as a HD model is easy visual evaluation of neurodegeneration. Overexpression of mHtt in *Drosophila *leads to the formation of aggregates, neuronal death, and decreased survival [57]. In addition, fly models of HD reproduce symptoms such as motor dysfunction and impairment of cognitive abilities and memory [58]. In the body wall muscle cells of *C. elegans *expressing the polyQ tract fused to the yellow fluorescent protein (YFP), the formation of aggregates, cellular toxicity, and paralysis were directly correlated with the age and the number of Q repeats [59]. Both mentioned models are actively used for testing potential drugs against HD. However, these models, which are based on species distantly related to humans, cannot fully reproduce the clinical picture observed in HD patients. For example, expression of Htt fragments containing polyglutamine tracts with 88Q or 128Q in *C. elegans *resulted in significant neuronal dysfunction and touch insensitivity, without causing neuronal death [60].

**Fig. 3:**
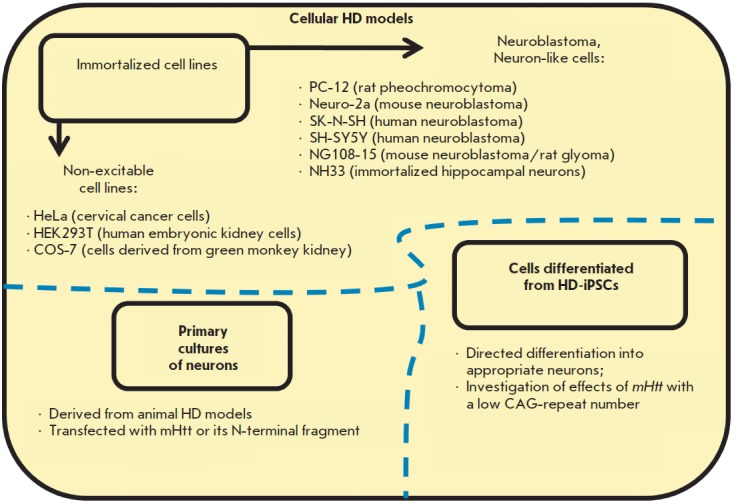
Cellular models of Huntington’s disease

A yeast model of HD is often used. For example, yeasts were used to demonstrate various pathological effects of mHtt aggregation: disruptions in endocytosis, tryptophan metabolism, cell cycle, and protein degradation [61-63].

There are also nongenetic animal models of HD, which are based on the use of chemical compounds (*Fig. 2*). For example, 3-nitropropionic acid and quinolinic acid are used as excitotoxic agents in animal models of HD. The first compound is a toxin that acts on mitochondria and induces neurotoxicity by irreversible inhibition of succinate dehydrogenase, the key respiratory chain enzyme responsible for the oxidation of succinate to fumarate. Quinolinic acid is an agonist of the N-methyl-D-aspartate receptor. The excitotoxicity induced by these compounds was studied in striatum slices, sagittal slices of the hippocampus [64], and in slices of the hippocampus of transgenic R6/2 mice [65].

It should be noted that many pathological manifestations of HD can be studied at the cellular level (*Fig. 3*). Cells can be transfected with both full-length mHtt and its fragments with a polyQ tract of a different length. For example, transfection of PC-12 cells with the first *mHtt *exon resulted in the nuclear localization of mHtt, changes in the morphology and expression of genes, and a lower rate of survival [66].

A large number of immortalized cell lines modeling HD have been generated, but not all pathological manifestations of HD can be revealed by these models. For this reason, primary neuronal cultures derived from transgenic mouse models of HD [67-69] or neurons isolated from wild-type animals and transfected with a vector for the expression of mHtt or its fragment have been used quite often [16].

An interesting and promising model of HD may be corticostriatal slices of a rat brain which are transfected with constructs expressing human mHtt. This model has an advantage over simple cellular models, because it maintains permanent cell-cell interactions, which is important in studying HD pathogenesis [70]. This model may be used to study the effect of potential therapeutic agents effective in HD.

One of the most advanced and promising approaches to the modeling of HD and other neurodegenerative diseases is the use of patient-specific induced pluripotent stem cells (HD-iPSCs) that endogenously express mutant huntingtin. Protocols for differentiating iPSCs into a phenotype similar to the phenotype of striatal medium spiny neurons (MSNs) [71-73], the cells most affected in HD, have been developed. One of the advantages of HD-iPSCs is the opportunity to study the pathological processes associated with the expression of mHtt with a short polyQ tract [73], which usually does not cause pathological changes in other models.

The expression of genes and proteins in HD-iPSCs differed from that in the controls; changes in proteostasis, neuronal development, intracellular transport, RNA metabolism, and cellular metabolism were observed [74]. In addition, the degree of expression disturbance was directly correlated with the polyQ tract length. Neurons differentiated from HD-iPSCs had a disease-associated phenotype, including electrophysiological changes and changes in metabolism, cell adhesion, and cellular toxicity. Cells containing the longest polyQ tract were the most sensitive to stress: e.g., to the absence of the brain-derived neurotrophic factor (BDNF) in the cell medium. Studies of neurons differentiated from HD-iPSCs revealed changes in the lysosomal activity [73, 75], mitochondrial fragmentation [76], and transcriptional repressor activity [77].

Another area of HD-iPSC application is cell transplantation for replacing diseased cells. Neuronal precursors differentiated from iPSCs were implanted into rat HD models. In this case, restoration of normal behavior was observed [78]. HD-iPSC-derived neural precursors were found to similarly restore the population of GABAergic striatal neurons and normalize the behavior of rats; however, the transplanted cells began to exhibit pathological properties at later stages [78], emphasizing the need for preliminary genetic correction in autologous transplantation.

## CALCIUM DYSHOMEOSTASIS IN HD

Calcium-signaling disruptions are characteristic of various neurodegenerative diseases, such as HD, Alzheimer’s disease, Parkinson’s disease, and amyotrophic lateral sclerosis [16, 79-81]. In animal HD models, which were created using genetically delivered mHtt or induced by 3-nitropropionic acid (3-NPA), calcium-signaling disruptions were shown to be a hallmark of HD.

**Fig. 4:**
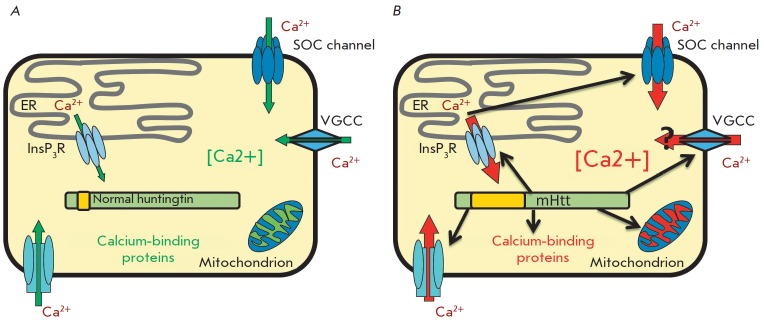
Calcium signaling disturbances in cells expressing mHtt. *A *– major pathways of calcium homeostasis regulation in normal cells. Green arrows indicate main calcium flows in health. *B *– the effect of mHtt expression on calcium homeostasis in the cell. Red arrows denote main impaired or potentially impaired calcium flows in HD. Changes in mitochondrial membrane permeability and disturbances in the expression and function of calcium-binding proteins are also shown in red. Black arrows indicate the calcium-signaling mechanisms affected by mHtt expression. ER – endoplasmic reticulum, NMDAR – N-methyl D-aspartate receptor, VGCC – voltage-gated calcium channel, SOC channel – store-operated calcium channel, InsP3R – inositol-1,4,5-trisphosphate receptor.

MHtt affects calcium signaling in the cell in many directions, including interactions with calcium-binding proteins and mitochondrial membranes, regulation of calcium influx from the extracellular medium, and release of calcium from intracellular stores (*Fig. 4*).

The main participants in neuronal calcium signaling include the calcium-binding proteins activated by binding to Ca^2+^ and regulating the free Ca^2+^ level, proteins exporting Ca^2+^ from the cytosol to the extracellular medium (plasma membrane ATPase, Na^+^/Ca^2+^ exchangers) or organelle cavity (SERCA), and the calcium channels involved in Ca^2+^ delivery to the cytoplasm [82, 83].

MHtt directly interacts with calcium-binding proteins [84], which may lead to an increase in the intracellular Ca^2+^ concentration and dysfunction of the proteins [85]. In particular, interaction between mHtt and calmodulin was found to occur in large molecular-weight-protein complexes [84], and disturbance of this interaction had a neuroprotective effect [85, 86]. One of the causes of an adverse effect of a prolonged increased Ca^2+^ level in the cytosol is the activation of calpain, a Ca^2+^-activated cysteine protease the action of which is almost irreversible. Calpain destroys cytoskeletal proteins and other perimembrane proteins. In a *Drosophila *model of HD, inhibition of calpain was shown to prevent the aggregation and toxicity of mHtt, stimulating autophagy. Overexpression of a calpain inhibitor, calpastatin, increases the number of autophagosomes and has a positive effect on mouse models of HD, which makes this process appropriate for developing approaches to HD therapy [87].

It is important to note that calcium-signaling disturbance in HD occurs at the transcription level, because mHtt fragments change the expression of some calcium homeostasis genes both in mouse models and in HD patients [6, 88]. Genomic studies conducted in various HD models have revealed significant differences in the mRNA levels of the genes encoding the proteins involved in intracellular Ca^2+^ regulation, including calcium-binding proteins such as parvalbumin, calmodulin, calbindin, and hypocalcin, as well as ryanodine receptor type 1, the inositol trisphosphate receptor (InsP3R1), and different subunits of voltage-gated calcium channels (VGCCs) [88-91]. In particular, the level of mRNA in the sarco-endoplasmic reticulum-associated ATP2A2 calcium pump (SERCA2) was reduced in peripheral blood mononuclear cells in HD [92].

Recently, a number of genes encoding calcium-signaling proteins the expression of which was disturbed in neurons differentiated from HD-iPSCs were identified using an analysis of gene ontology categories [73].

Transcription disturbances can be additionally enhanced by calcium-dependent control mechanisms. This may occur due to abnormal calcium-dependent regulation of the activity and stability of transcription factors, as well as changes in the functions of some calcium-binding proteins: e.g., the transcriptional repressor DREAM (downstream responsive element antagonist modulator), which is translocated to the nucleus in response to an increase in [Ca^2+^] in the cytosol [93, 94], as well as the cofactor LMO4, the activity of which is induced by Ca^2+^ influx via VGCC [95].

Also, the activity of glutamate receptors increases in HD, which leads to a significant calcium influx into the cytosol via the plasma membrane (PM), neuronal disturbances, and cell death. A relationship between polyglutamine expansion and neuronal sensitivity to glutamate-mediated excitotoxicity has been established [96]. Increased calcium influx into the cytosol via NMDA receptors (NMDARs) is associated with the potentiating effect of mHtt on the transport and incorporation of NMDAR into PM [97]. In this case, differences in the expression level and subunit composition of NMDARs in different cells may be one of the causes for the selective death of MSNs in HD [98]. Pharmacological inhibition of NMDARs exerted a neuroprotective effect on a primary culture of MSNs of HD mouse models [99, 100]. It should also be noted that YAC128 mice were characterized by an increased expression of the extra-synaptic NMDAR, which resulted in disruption of the p38 MAPK and CREB signaling pathways, as well as dysfunction and atrophy of the striatum [101].

Also, mHtt was shown to affect VGCC by binding directly to the auxiliary α2/δ subunit of VGCC [102]. The association of the N-terminal domain of huntingtin (both mutant and normal) with the pore-forming CaV2.2 subunit of N-type VGCC leads to a displacement of the syntaxin 1A that negatively regulates the channel and, as a result, to an increase in the activity of N-type VGCC [103]. This example indicates the potential physiological functions of cleavage of the N-terminal fragment from normal huntingin, whereas further research is needed to understand the role of N-type VGCC in the disease. At the same time, a potential hyperfunction of VGCC in HD is confirmed by the results obtained in *Drosophila*, which demonstrate that removal of Dmca1D (the L-type VGCC channel in *Drosophila*) leads to decreased photoreceptor neurodegeneration [104].

Overexpression of mHtt fragments in striatal neuronal precursor cells (Q7/7) resulted in a significant decrease in [Ca^2+^] in the endoplasmic reticulum (ER), while [Ca^2+^] in the cytosol remained the same as in the controls [105]. Application of cyclopiazonic acid induced an increased release of Ca^2+^ from the ER to the cytosol in a striatal cell line derived from knock-in mouse embryos expressing mHtt with 111Q [106]. At the same time, expression of mHtt in PC-12 cells did not lead to statistically significant changes in the ER calcium level [91].

MHtt (but not wild-type Htt) was shown to directly interact with the C-terminal region of InsP3R1, increasing its sensitivity to InsP3 [107], thereby promoting the outflow of Ca^2+^ from the ER. An important role of InsP3R1 in polyglutamine expansion-induced neurotoxicity was experimentally confirmed in a primary culture of MSNs from a HD mouse model [99, 102] and in *Drosophila *[108]. Also, a peptide that disrupts the interaction between mHtt and InsP3R1 was found to exert a neuroprotective effect on MSN cells from a HD model [109]. In addition, inhibition of *InsP3R *gene expression reduces mHtt aggregation [110], which emphasizes the importance of the interaction of two proteins in the pathogenesis of HD.

MHtt that interacts with InsP3R1 and, thereby, affects the ER Ca^2+^ level may disrupt the functions of store-operated calcium (SOC) channels. These channels are activated in response to a decrease in the calcium concentration in intracellular calcium stores, the most common of which is the ER. Thus, the activation of InsP3R1 will result not only in store depletion, but also in subsequent store-operated calcium entry via the PM. It is important to note that disruption of SOCE (SOC Entry) has been established in many neurodegenerative diseases, including Alzheimer’s disease, spinocerebellar ataxia, and HD [80, 111-113].

SOCE disruption may be caused by a change in the level of STIM1/2 proteins containing EF-hand domains and acting as calcium sensors in the ER lumen. These changes can be caused by the impaired proteasomal degradation that occurs in neurodegeneration [114].

A significant increase in SOCE was found in SK-N-SH neuroblastoma cells expressing mHtt 138Q [113]. It was suggested that the significant increase in SOCE in cell models of HD was mediated not by changes in the properties of SOC channels but by an increase in their number; however, it should be noted that no direct experimental evidence of this hypothesis was provided.

A significant increase in SOCE was also found in SK-N-SH cells expressing not full-length mHtt, but its N-terminal fragment. Additionally, the STIM1 protein was shown to be required for SOCE activation. Suppression of STIM1 was accompanied by a decrease in SOCE, and detected currents might be divided into two types: high and low reversal potentials, which implies competition of at least two types of SOC channels for interaction with STIM1 [115]. The data indicating that at least two different proteins mediate calcium entry by the store-operated mechanism was also obtained in HD models: Neuro-2a mouse neuroblastoma cells and a primary culture of mouse striatal neurons [16]. Using patch-clamp and RNA interference, the authors found that the pore-forming proteins Orai1 and TRPC1 together maintain SOCE in cells that express an N-terminal fragment of mHtt with 138Q, which may be explained by the existence of a heteromeric channel containing subunits of Orai1 and TRPC1 [16]. This heteromeric channel was hypothesized as early as 2007 [116], but no experimental evidence confirming this idea was presented. At the same time, calcium entry via Orai1-formed channels was shown to be necessary for the incorporation of TRPC1 proteins into the PM [117]. Therefore, it may be assumed that TRPC1 proteins largely contribute to the amplitude of store-operated currents in a Neuro-2a cell model of HD, which is confirmed by a dramatic current drop upon TRPC1 suppression. However, upon Orai1 suppression, a significant reduction in the SOCE amplitude was also observed, which may now be explained not only by a decrease in the current through Orai1, but also by a decreased TRPC1-mediated current component due to a disruption of TRPC1 traffic to the plasma membrane [16]. The importance of TRPC1 in the pathogenesis of HD is also confirmed by data demonstrating that TRPC1 suppression by a short interfering RNA has a significant protective effect on MSNs of YAC128 mice in a model of glutamate-induced apoptosis. In this case, suppression of TRPC1 in the neurons of wild-type mice had practically no effect on glutamate-mediated cell death [111].

Expression of the N-terminal fragment of mHtt in a primary culture of MSNs also results in abnormally large store-operated calcium entry into the cytosol [16]. These results are confirmed by measurements of the intracellular calcium concentration using a calcium probe, FURA-2, in MSN cells isolated from YAC128 mice [111]. Furthermore, the effect of a NF-κB signaling pathway inhibitor, EVP4593, on these cells was studied. There is a close relationship between activation of NF-κB and store-operated calcium entry [118, 119]. NF-κB is able to bind to the *Htt *gene and enhance the activity of its promoter in mouse striatal neurons [24]. MHtt can also bind to one of the key enzymes of the NF-κB signaling pathway, IKK, thereby increasing its activity [120].

EVP4593 was shown to reversibly reduce abnormally large SOCE to control values both in SK-N-SH cells expressing mHtt with 138Q and in MSNs of YAC128 mice [111]. EVP4593 exerted a similar effect on MSN cells expressing an N-terminal fragment of mHtt [16]. Now, EVP4593 is proven to act as an inhibitor of SOCE necessary for the initial stages of the NF-κB signaling pathways; however, the molecular target of EVP4593 remains unknown.

It should be noted that EVP4593 has a high therapeutic potential, because it exerts a neuroprotective effect in glutamate-induced apoptosis of MSNs from YAC128 mice and induces a positive effect in motor assays in fly models of HD [111]. Cytofluorimetric measurements demonstrated that incubation of Neuro-2a cells (HD model) with EVP4593 results in increased survival of the cells [16].

The published data suggest that the neuroprotective effect of EVP4593 is based on a negative feedback present in mHtt influencing the cell. Since NF-κB is able to bind directly to *Htt *and enhance the activity of its promoter [24], and EVP4593 inhibits NF-κB signal transduction, a potential result of EVP4593 application may be a decreased mHtt expression and, as a consequence, a decrease in the toxic functions of mHtt. Nevertheless, additional research is required to confirm this idea.

Of special interest is a study of the effect of mHtt expression on SOCE which was performed in HD-specific human neurons differentiated from iPSCs and expressing mHtt with a low Q number in the tract. Despite the fact that the polyglutamine tract of mHtt in this HD model contained only 40–47Q, which was close to the normal value, changes in SOCE were as significant as those in other models with a tract length exceeding 100Q [73]. In this case, EVP4593 decreased the SOCE amplitude both in pathology and in controls and likewise had a neuroprotective effect upon exposure to the proteasome inhibitor MG132 [73].

**Fig. 5:**
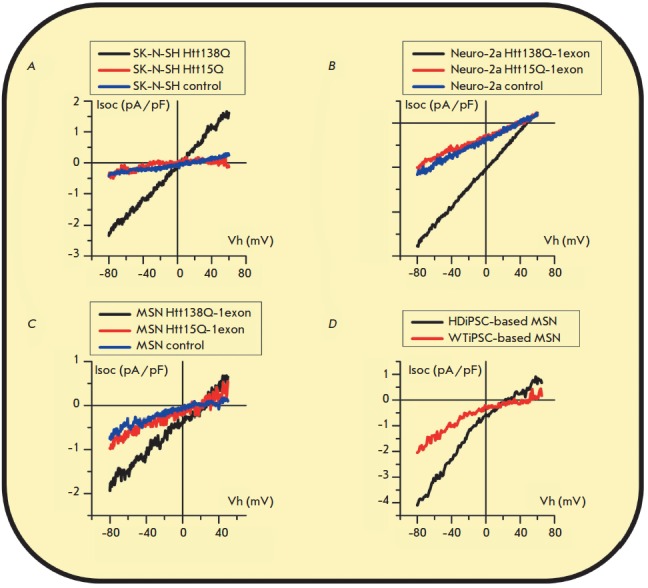
Abnormal increase in store-operated calcium entry in cellular models of Huntington’s disease. Mean current– voltage curves presented at maximum level of current development and normalized to cell capacitance, which represent store-operated calcium entry in SK-N-SH human neuroblastoma cells expressing full-length huntingtin 138Q, 15Q, or an empty control vector (*A*) [111]; in Neuro-2a mouse neuroblastoma cells expressing the first exon of huntingtin containing 138Q, 15Q, or an empty control vector (*B*) [16]; in a primary culture of mouse striatal MSNs expressing the first exon of huntingtin containing 138Q, 15Q, or an empty control vector (*C*) [16]; in human neurons differentiated from HD-specific iPSCs or wild-type iPSCs (*D*) [73].

In general, the conducted studies demonstrate that SOCE abnormalities are systemic and occur in various cellular models of HD (*Fig. 5*) [16, 73, 111]. This fact may indicate that SOCE abnormalities precede other pathological processes in HD and, probably, are of the central mechanisms underlying neurodegeneration. Thus, SOCE may be considered a promising target for the development of approaches to HD therapy and the data obtained in various cellular models may be used for the development of EVP4593-based drugs.

An increase in SOCE is supposed to directly affect the ability of mitochondria to store Ca^2+^ , since mitochondria are located in immediate vicinity to the site of ER Ca^2+^ release [121]. The mitochondrion is one of the main regulators of the intracellular Ca^2+^ level. A significant increase in [Ca^2+^] in the cytosol, in the immediate vicinity of the mitochondrion, is accompanied by the activation of the low affinity mitochondrial Ca^2+^ uniporter (MCU) mediating Ca^2+^ influx into the matrix. The mitochondria release Ca^2+^ via the Na^+^/Ca^2+^ exchanger [122] or, in the case of calcium overload, via megapores (PTP), the activation of which leads to a membrane potential jump, rupture of the outer membrane, and release of cytochrome C and caspases, which results in apoptotic cell death [123, 124]. The involvement of mitochondrial dysfunction in the pathogenesis of HD is confirmed, in particular, by the fact that 3-nitropropionic acid, which is used as an inhibitor of the mitochondrial respiratory chain complex II, causes impairments typical of HD [125]. An additional piece of evidence of the important role of mitochondria in the pathogenesis of HD is the neuroprotective effect of mitochondrial membrane permeability inhibitors, which has been demonstrated in both cellular and animal models [99, 126].

Expression of mHtt was also accompanied by defects in mitochondria morphology. In a cell line derived from knock-in mouse embryos expressing mHtt with 111Q, mitochondria are more prone to fragmentation because abnormal [Ca^2+^] in the cytosol promotes an increase in the activity of a calcium-dependent phosphatase, calcineurin, dephosphorylating (and, thereby, activating) the Drp1 protein responsible for mitochondrial division. Finally, enhanced mitochondrial fragmentation promotes cell apoptosis [106].

Impairment of Ca^2+^ buffering and calcium metabolism in mitochondria was detected at both early and late stages of HD, which indicates the key role of these impairments in the pathogenesis of HD. Mitochondria isolated from the brain cells of HD patients and from the cells of HD mouse models were more sensitive to Ca^2+^ stress and tended to form megapores [127, 128]. Similar results were obtained later in an immortalized line of striatal neuronal precursor cells derived from knock-in KI-HdhQ111 mice [129]. However, the susceptibility of mitochondria to calcium stress was reproduced not in all experimental models. For example, striatial mitochondria isolated from knock-in mice expressing different mHtt variants (80, 92, or 111Q), R6/2 mice, and YAC128 mice were equally, and in some cases even less, susceptible to Ca^2+^ stress than the control wild-type samples [130, 131]. In addition, the sensitivity of mitochondria to Ca^2+^ stress in some HD models decreased proportionally to the age and polyQ tract length [130], which suggests the presence of protective compensatory mechanisms. A recent study of isolated mitochondria and striatal neurons in R6/2 mice has also revealed the absence of respiratory chain dysfunction and increased mitochondrial sensitivity to calcium stress [132]. Therefore, the role of mitochondria in the pathogenesis of HD remains controversial. Further research is needed to elucidate the molecular mechanisms of the disease.

## CONCLUSION

Despite the long history of HD research, the issues of manifestation, simulation, and investigation of the molecular basis of the disease remain topical. This review has described the cellular and animal models widely used in investigations of the pathological processes in HD, as well as in screening for potential drugs. Of particular interest are models based on endogenous expression of mutant huntingtin in neurons differentiated from patient-specific iPSCs. The analysis of recent publications indicates that abnormal calcium signaling is one of the central links that mediate the development of the pathology and lead to neuronal death. One of the most important elements of calcium signaling, which is impaired in HD, is the store-operated calcium entry, whose pathological increase was demonstrated in many of the models described in this review. It is likely that an abnormal ER calcium level, together with coupled excessive store-operated calcium entry via the PM, may affect the mitochondria that activate the process of cell death, being unable to store excessive calcium.

In summary, it should be noted that the investigation of neurodegeneration is a research field that is developing intensively, which gives hope that a complete picture of neurodegeneration processes could be built and that new drugs effective against HD, Alzheimer’s disease, Parkinson’s disease, and other pathologies would be developed.

## Abbreviations

HD: Huntington’s disease
PM: plasma membrane
ER: endoplasmatic reticulum
[Ca2+]: calcium concentration
(HD)iPSCs: (HD specific) induced pluripotent stem cells
InsP3: inositol-1,4,5-trisphosphate;
InsP3R: inositol-1,4,5-trisphosphate receptor
(m)Htt: (mutant) huntingtin
Q: glutamine residue
MSNs: striatal medium spiny neurons
SOC(E): store-operated calcium (entry)
